# Concordance of vaccination status and associated factors with incomplete vaccination: a household survey in the health district of Segou, Mali, 2019

**DOI:** 10.11604/pamj.2021.40.102.29976

**Published:** 2021-10-14

**Authors:** Sidiki Sangaré, Oumar Sangho, Lancina Doumbia, Hannah Marker, Yeya dit Sadio Sarro, Housseini Dolo, Nouhoum Telly, Issa Ben Zakour, Hadji Mamadou Ndiaye, Moussa Sanogo, Fanta Sangho, Niélé Hawa Diarra, Aboubacar Sangho, Fatoumata Bintou Traoré, Baba Diallo, Cheick Abou Coulibaly, Sadou Ongoiba, Lamine Diakité, Seydou Doumbia

**Affiliations:** 1 Health District of Macina, Macina, Mali,; 2 Department of Education and Research of Biological and Medical Sciences, Faculty of Pharmacy, University of Sciences, Techniques and Technologies of Bamako, Bamako, Mali,; 3 Department of Education and Research in Public Health and Specialties, Faculty of Medicine and Dentistry, University of Sciences, Techniques and Technologies of Bamako, Bamako, Mali,; 4 Department of International Health, Johns Hopkins Bloomberg School of Public Health, Baltimore, Maryland, USA,; 5 Health District of Segou, Segou, Mali,; 6 Regional Directorate of Health and Public Hygiene, Segou, Mali,; 7 Regional Directorate of Health and Public Hygiene, Kidal, Mali,; 8 Department of Education and Research of Pharmaceutical Sciences, Faculty of Pharmacy, University of Sciences, Techniques and Technologies of Bamako, Bamako, Mali,; 9 National Institute of Public Health, Bamako, Mali,; 10 University Hospital Center for Odonto-Stomatology (CHU-CNOS), Bamako, Mali,; 11 Community Health Center of Pélengana South, Segou, Mali

**Keywords:** Incomplete vaccination, concordance, associated factors, children, Segou, Mali

## Abstract

**Introduction:**

the region of Segou recorded 36.8% of children were incompletely vaccinated in 2018. In 2019, the district of Segou was one of the districts with the lowest vaccination coverage in the region, with 85.1% coverage for the three doses of the pentavalent vaccine and 85.4% for the measles vaccine. This study was initiated to better understand this low vaccination coverage, in the absence of specific studies on vaccination coverage in the district of Segou.

**Methods:**

a prospective cross-sectional study was conducted from May to August 2020 with 30 clusters. We performed Kappa coefficient, bivariate, and multiple logistic regression analysis.

**Results:**

findings showed that 18.46% (101/547) [15.44-21.93] of children were incompletely vaccinated. Mothers correctly reported the vaccination status of their children in 67.30% of cases (Kappa coefficient). Uneducated (OR[IC95%]=2.13[1.30-3.50]), living in rural area (OR[IC95%]=2.07[1.23-3.47]), lack of knowledge of Expanded Program on Immunization (EPI) target diseases (OR[IC95%]=2.37[1.52-3.68]), lack of knowledge of vaccination schedule (OR[IC95%]=3.33[1.90-5.81]) and lack of knowledge of the importance of vaccination (OR[IC95%]=3.6[2.35-6.32]) were associated with incomplete vaccination. In multivariate analysis, uneducated (ORa[IC95%>]=1.68[1.004-2.810]) and lack of knowledge of the importance of vaccination were associated with incomplete vaccination (ORa[IC95%]=3.40[2.049-5.649]).

**Conclusion:**

findings showed a good concordance of the vaccination status. Living in a rural area, no education, lack of the knowledge of EPI target diseases, lack of the knowledge of vaccination schedule and lack of knowledge of the importance of vaccination were associated with incomplete vaccination.

## Introduction

The Expanded Program on Immunization (EPI) is a public health intervention that aims to immunize children around the world to prevent and to reduce disability and death due to vaccine-preventable diseases [1]. Immunization saves the lives of 2.5 to 3 million children each year [1,2]. In 2018, around 20 million children worldwide did not receive lifesaving vaccines such as those against measles, diphtheria, and tetanus and most of those children live in low-income countries and countries with conflict [3]. Factors associated with these inequalities are identified such us maternal literacy, household residence, sex of the child, or socio economic status of the parents [4].

In Mali, despite the more than 30 years of implementing the EPI and the creation of hundreds of community health centers whose primary objective is prevention (mainly through vaccination), 41% of children aged 12-23 were incompletely vaccinated in 2018 [5]. The same year, in the region of Segou, 36.8% of children aged 12-23 months were incompletely vaccinated [5]. In 2019, the health district of Segou was one of the districts in the Segou region with the lowest vaccine coverage, with 85.1% coverage for the three doses of the pentavalent vaccine, and 85.4% for the measles vaccine [6]. These vaccination coverage rates are low compared to the target of 95% of the 2017-2021 EPI plan [7]. Unlike other health districts that had low vaccine coverage, the health district of Segou was not exposed to insecurity due to intercommunity and religious conflict, which could explain these low vaccine coverage levels. To better understand these low vaccination coverage rates, in the absence of specific studies on vaccination coverage in the health district of Segou, we initiated this study. The objective was to assess the vaccination coverage, concordance, and the associated factors in children aged 12-23 months in the Malian health district of Segou in 2019.

## Methods

**Study framework**: the study took place in the health district of Segou, located in the center of the Segou region. In 2019, its population was 608,707 inhabitants. The vaccination target was estimated at 4% of the population, i.e. 24,348, of which 40.40% are reached by the advanced strategy (travel of the vaccinator to vaccinate children who live more than 5 km from the health center). The district has one referral health center (CSRéf) and 36 health areas (five urban and 31 rural). Each health area, in addition to the CSRéf, holds at least one vaccination session in a fixed location each week. Rural health areas organize advanced vaccination strategy sessions according to the number of villages to be vaccinated.

**Type and period of study**: this was a prospective cross-sectional community-based study that took place over a period of four months, from May 1^st^ to August 31^st^, 2020.

**Study population**: included in this study were the mothers of children aged 12 to 23 months living in the Segou health district for at least one year. Mothers of children aged 12 to 23 months who were absent at the time of the survey, and those who declared their children were vaccinated but could not show the vaccination card or the children´s name were not verifiable in the vaccination register, were not included in this study.

**Sample size**: the sample size was calculated using the Epi info software version 7.2.3.1 using the population survey formula (Fleiss). The following parameters were taken into account in calculating the sample size: confidence level (95%), number of clusters (30), frequency of children incompletely vaccinated in the region (36.8%), and cluster effect (1.5). The minimum sample size was estimated to 540 participants to be surveyed, which we rounded up to 600 people, which represented 20 people per cluster.

**Sampling techniques**: we conducted a cluster survey. In the district, 30 clusters were selected using the cumulative population size method among 405 villages and neighborhoods. In each cluster, households were randomly selected by spinning a pen in the middle of the cluster. Then we followed the direction of the tip of the pen and entered the first household in that direction to investigate. Once in the household, all mothers of eligible children were surveyed and we moved on to the next household. Thus the evolution was made step by step until completing the size of the cluster.

**Data collection**: data were collected using a questionnaire tested beforehand. The questionnaire was designed through the adaptation of previous studies [1,8]. Questions were asked on vaccination status of the child, socio-demographic characteristics (i.e. age, marital status, residence, education, number of children, activities generating income) and knowledge about vaccination (i.e. vaccination schedule; EPI target diseases and the importance of vaccination). The vaccination status of the child was then checked on the card or in the vaccination register. From these questionnaires, data were entered into a database on SPSS software.

**Analysis**: data were analyzed using the Statistical Package for Social Sciences (SPSS 25.0) and EPI Info version 7.2.3.1. Vaccination status was the dependent variable. Age was categorized in three groups: less than 25, 25-33, and 34 and more. We performed a descriptive analysis, and bivariate and multivariate logistic regression. Kappa coefficient was used to calculate concordance between vaccination status according to mother declaration and the vaccination status checked on vaccination card or register. Sensitivity and specificity were evaluated. Sensitivity was defined as the probability to find an incompletely vaccinated child when the mother said the child was not completely vaccinated. Specificity was defined as the probability to find a completely vaccinated child when the mother said the child was completely vaccinated. Bivariate analysis were conducted and Odds Ratios (OR) were presented with a p value of 0.05 as significant level. Variables that had a significant association during the bivariate analysis were selected for the multivariate (global) model. Multiple logistic regression was performed and adjusted Odds Ratios (ORa) were reported with a p value of 0.05 as significant level (backward elimination; step by step).

### Definition of concepts

**Vaccination Status: fully or completely vaccinated**: a child who received one dose of Bacillus Calmette-Guérin (BCG) vaccine, three doses of Oral Polio Vaccine (OPV) (excluding OPV given at birth), three doses of pentavalent vaccine, three doses of Pneumococcal Conjugate Vaccine (PCV13), one dose of measles vaccine (VAR), one dose of yellow fever vaccine (AAV) and one dose of meningococcal A vaccine (MenAfriVac) before 12 months of age.

**Incompletely vaccinated**: a child who started the vaccination schedule and did not complete it before 12 months of age.

**Knowledge of the target diseases of the vaccination**: being able to name at least two diseases of the EPI.

**Knowledge of the vaccination schedule**: being able to cite the five appointments of the vaccination schedule.

**Mother's education**: educated (if the mother got school, at least the primary level) uneducated (if the mother had no education, never got school).

**Vaccination in advanced strategy**: vaccination of children who live more than 5 km away from the health center. Health worker go to such village for vaccination.

**Number of appointments: Number of appointments**: there are five appointments as follow: First: child who received BCG vaccine before 12 months of age. Second: child who received the first doses of pentavalent vaccine, OPV, PCV13 before 12 months of age. Third: child who received the second doses of pentavalent vaccine, OPV, PCV13 before 12 months of age. Fourth: child who received the third dose of pentavalent vaccine, OPV, PCV13 before 12 months of age. Fifth: child who received one dose of BCG vaccine, three doses of OPV, three doses of pentavalent vaccine, three doses of PCV13, one dose of measles vaccine (VAR), one dose of yellow fever vaccine (Anti Amaril Vaccine (AAV)) and one dose of meningococcal A vaccine (MenAfriVac) before the age of 12 months.

**Ethical considerations**: this study has been approved by the administrative authorities, the chief of the health district of Segou and all the community health center authorities of the Segou health district. The informed consent of the mothers was obtained verbally after explanations of the study objectives and procedures. This study had no risk for participants. Participants could withdraw at any time without consequences. During this survey, confidentiality was guaranteed by not reported any data linked to the participants.

## Results

Of the 600 children, 53 had never been vaccinated and were excluded from the analysis. The remaining 547 (91.17%) was analyzed. The frequency of incompletely vaccinated children was 18.46% (101/547) [15.44-21.93]. The frequency of completely vaccinated children was 81.54% (446/547) [78.07-84.56].

**Factors associated to vaccination status**: among the 547 participants, 60.15% were aged 25 to 34 years old. Among the participants, 63.07% were uneducated and 67.46% pf them lived in rural area. Uneducated (OR[IC95%]=2.13[1.30-3.50]), living in rural area (OR[IC95%]=2.07[1.23-3.47]), lack the knowledge of EPI target diseases (OR[IC95%]=2.37[1.52-3.68]), lack of knowledge of vaccination schedule (OR[IC95%]=3.33[1.90-5.81]) and lack of the importance of vaccination (OR[IC95%]=3.86[2.35-6.32]) were associated with incomplete vaccination. Our results showed that 35.47% of the surveyed mother gave birth at home. Approximatively, 51.19% of the children enrolled in the survey were female (Table 1).

**Table 1:** sociodemographic, economic characteristics and mothers' knowledge of vaccination in the health district of Segou in 2019

Factors	Incomplete vaccination n(%)	Complete vaccination n (%)	Total (%)	OR[IC95%]	p
**Age (in years)**					
< à 25	24(17.91)	110(82.09)	24.50	1	
25-34	62(18.84)	267(81.16)	60.15	0.94[0.56-1.58]	0.815
≥ 34	15(17.86)	69(82.14)	15.35	1.004[0.49-2.04]	0.992
**Educated**					
Yes	24(11.88)	178(88.12)	36.93	1	
No	77(22.32)	268(77.68)	63.07	**2.13[1.30-3.50]**	**0.003**
**Residence**					
Urban	21(11.80)	157(88.20)	32.54	1	
Rural	80(21.68)	289(78.32)	67.46	**2.07[1.23-3.47]**	**0.006**
**Income-generating activity**					
Yes	74(17.96)	338(82.04)	75.32	1	
No	27(20.00)	108(80.00)	24.68	1.14[0.70-1.87]	0.596
**Knowledge of EPI target diseases**					
Yes	52(14.02)	319 (85.98)	67.82	1	
No	49(27.84)	127(72.16)	32.18	**2.37[1.52-3.68]**	**0.00009**
**Knowledge of the vaccination schedule**					
Yes	76(15.80)	405(84.20)	87.93	1	
No	25(38.46)	40(61.54)	12.07	**3.33[1.90-5.81]**	**0.00001**
**Knowledge of the importance of vaccination**					
Yes	65 (14.29)	390(85.71)	83.18	1	
No	36(39.13)	56(60.87)	16.82	**3.86[2.35-6.32]**	**10-8**
**Birth location**					
Health center	57(16.15)	296 (83.85)	64.53	1	
Home	44(22.68)	150(77.32)	35.47	1.52[0.98-2.36]	0.059
**Gender of the child**					
Male	47(17.60)	220(82.40)	48.81	1	
Female	54(19.29)	226 (80.71)	51.19	1.12[0.73-1.72]	0.612
**Birth rank**					
One or two	32(17.98)	146(82.02)	32.54	1	
At least three	69(18.70)	300(81.30)	67.46	1.04[0.66-1.66]	0.838

**Concordance of immunization status**: the sensitivity of the declaration of mothers on the vaccination status of children (Se) was 56.43% and the specificity of the declaration of mothers on the vaccination status of children (Sp) was 99.77%. The Kappa reproducibility coefficient was 67.30% (Table 2).

**Table 2:** distribution of the vaccination status of children declared by mothers and that verified in the vaccination materials

Variable		Vaccination status on the card/register		Total
		Incomplete	Complete	
**Vaccination status according to mother**				
	Incomplete	57	1	**58**
	Complete	44	445	**489**
	**Total**	**101**	**446**	**547**

Kappa coefficient = 67.30% with ln(OR)[IC95%]=6.37[4,36-8,36], p=0,0001. Sensitivity = 56.43% Specificity = 99.77%

**Factors associated with incomplete immunization**: Using multivariate analysis, uneducated and lack of the importance of vaccination were associated with incomplete vaccination given Adjusted Odds Ratios of ORa[IC95%]=1.68[1.004-2.810] and ORa[IC95%]=3.40[2.049-5.649] respectively (Table 3).

**Table 3:** factors associated with incomplete vaccination in children aged 12-23 months in the health district of Segou in 2019 (multivariate analysis)

Factors	Incomplete vaccination (%)	Complete vaccination (%)	ORa[IC95%]*	p
**Educated**				
Yes	23.76	39.91	1	
No	76.24	60.09	1.68 [1.004-2.810]	0.048
**Knowledge of the importance of vaccination**				
Yes	64.36	87.44	1	
No	35.64	12.56	3.40 [2.049-5.649]	0.0001

* ORa=Adjusted Odds Ratio

## Discussion

**Vaccination coverage**: results showed that more than 80% of children were completely vaccinated. This vaccination coverage completeness was high compared to the 52% of the Mali sixth Demographic and Health Survey (DHS), but low compared to the target of 95% of the 2017-2021 EPI plan [5,7]. The vaccination coverage found corroborates those of previous studies with 77.9% in Kouroussa, Guinea [9], 72.2% in Senegal [10] and 76.8% in Ethiopia [11]. Our vaccination coverage was greater than those found in Cameroon 64.3% [1] and in Ethiopia with 38.3% [12,13]. This difference could be explained by the differences between the data collection sources, based on the vaccination card and the vaccination register in our study versus the vaccination card and the verbal statement of mothers for the studies in Cameroon and in Ethiopia, with possibility of loss of information. Our vaccine completeness was lower than that of a study in Kaolack, Senegal [14]. This gap could be explained at the level of the health system. In the Segou health district, the vaccination was done using different strategies like fixed strategy (done at the health centers), advanced strategy (done in the village far from the health centers).

These strategies are not the same used in Kaolack, where vaccination takes place daily, using only the fixed strategy. This daily availability of vaccination services and its integration in the general health services could give many children the chance to complete their vaccinations. By comparing the information provided by the mothers on the vaccination status of the children with the information on the vaccination cards or in the vaccination register, we found a Kappa coefficient of reproducibility of 67.30%. This concordance shows that in more than 67% of the cases, the statements made by the mothers were correct. This match could be used as an argument for carrying out large-scale studies on the vaccination status of children only based on mother's reports. Our agreement was better than that of Adedire *et al*. in 2016 who found a rate of 33.6% in Nigeria [15].

### Factors associated with incomplete vaccination

**Residence and education**: living in rural area and being uneducated were associated with incomplete vaccination. Indeed, children whose mothers did not attend school and who live in rural areas had approximatively 3 times more risk of being incompletely vaccinated compared to children whose mothers were educated and live in urban areas. Previous studies conducted in Africa also found that literacy and education are factors associated with the vaccination status of children: OR[CI95%]=2.34[1.12, 4.47] in Nigeria [16,17], OR[CI95%]=1,84[1,22-2,77] in Ivory Coast [18], OR[CI95%]=0.56[0.33-0.95] in Malawi [19], OR[CI95%]=1.38[1.07 1.78] in Ethiopia [13], OR[CI95%]=2.2 [1.6; 3.1] in Togo [20,21], OR[CI95%]=18.4 [4.01-84.62] in Somalia [22]. The education leads to the intellectual development of women and the possibility of access to many channels of information on routine vaccination while enhancing their attendance of vaccination services.

**Knowledge of Vaccination**: lack of awareness of the importance of vaccination was associated with incomplete vaccination of children. Children whose mothers did not understand the importance of vaccination were more than 3 times more likely to be incompletely vaccinated than those whose mothers did understand the importance of vaccination. Other authors have also highlighted the association between knowledge of the importance of vaccination and vaccination status in Cameroon (OR[CI95%]=4,4[1,35-14,42]) [1], in Ethiopia (OR[CI95%]=1.9 [1.44-2.49]), (OR[CI95%]=2.24[1.68-2.98]) [8,12], in Nigeria (OR[CI95%]=2.4 [1.6-3.8]) [15], in Kenya (OR[CI95%]=2.21[1.22-3.98]= [23]. Knowledge of routine vaccination information by the community improves their adherence to the EPI.

**Limitations of the study**: rotateq and IPV were not included in the definition of full vaccination for this study due to an untimely shortage of Rotateq and the similarity between IPV and OPV. The dates of vaccine administration were not taken into account but rather vaccine completeness before 12 months. These limitations do not affect the validity of the study. However, they can be considered selection biases as the inclusion of Rotateq and IPV in the definition would reduce the number of participants is the study.

## Conclusion

Our findings showed a good concordance of the vaccination status as reported by the mother and the official vaccination document. Living in a rural area, no education, lack of the knowledge of EPI target diseases, lack of knowledge of vaccination schedule and lack of knowledge of the importance of vaccination were significantly associated with incomplete vaccination. The good agreement as reported by Kappa coefficient in this study could be used as justification for conducting large-scale study on vaccination status based on mothers' reports as proxy measure.

### What is known about this topic

- Low vaccination completeness in Africa;
- Factors associated with this vaccination incompleteness are multiple vary from one country to another.

### What this study adds

- Findings of this study show that not knowing the vaccination schedule, and the importance of the EPI are strongly associated to the incompleteness of the vaccination in the district of Segou;
- There is consistency between the vaccination status reported by the mothers and the information in the vaccination documents.

## References

[1] Ba Pouth SFB, Kazambu D, Delissaint D, Kobela M (2014). Couverture vaccinale et facteurs associés à la non complétude vaccinale des enfants de 12 à 23 mois du district de santé de Djoungolo-Cameroun en 2012. Pan Afr Med J.

[2] Organisation Mondiale de la Santé (2019). La santé et le bien-être: objectif 3 de développement durable.

[3] Organisation Mondiale de la Santé (2019). Vaccination.

[4] Organisation mondiale de la Santé (2016). State of inequality: childhood immunization.

[5] (2018-2019). Institut National de la Statistique (INSTAT), Cellule de Planification et de Statistique Secteur Santé-Développement. ICF, Enquête Démographique et de Santé au Mali.

[6] Direction Régionale de la Santé et de l´Hygiène Publique de Ségou (2020). Annuaire Statistique Sanitaire 2019 du Système Local d´Information Sanitaire de la Région de Ségou.

[7] Direction Nationale de la Santé (2017). Plan Pluri-annuel Complet (PPaC) 2017-2021 du Programme Elargi de la Vaccination du Mali.

[8] Ababu Y, Braka F, Teka A, Getachew K, Tadesse T, Michael Y (2017). Behavioral determinants of immunization service utilization in Ethiopia: a cross-sectional community-based survey. Pan Afr Med J.

[9] Ngwa W, Mupenda J, Haba B, Nanan-N´Zeth K, Bachy C, Pineda S (2019). Enquête de couverture vaccinale multi antigénique Préfecture de Kouroussa.

[10] Mbengue MAS, Mboup A, Ly ID, Faye A, Camara FBN, Thiam M (2017). Vaccination coverage and immunization timeliness among children aged 12-23 months in Senegal: a Kaplan-Meier and Cox regression analysis approach. Pan Afr Med J.

[11] Legesse E, Dechasa W (2015). An assessment of child immunization coverage and its determinants in Sinana District, Southeast Ethiopia. BMC Pediatr.

[12] Lakew Y, Bekele A, Biadgilign S (2015). Factors influencing full immunization coverage among 12-23 months of age children in Ethiopia: evidence from the national demographic and health survey in 2011. BMC Public Health.

[13] Tamirat KS, Sisay MM (2019). Full immunization coverage and its associated factors among children aged 12-23 months in Ethiopia: further analysis from the 2016 Ethiopia demographic and health survey. BMC Public Health.

[14] Seck I, Diop B, Leyé MM, Mbacké Mboup B, Ndiaye A, Seck PA (2016). Déterminants sociaux de la couverture vaccinale de routine des enfants de 12 à 23 mois dans la région de Kaolack, Sénégal. Sante Publique.

[15] Adedire EB, Ajayi I, Fawole OI, Ajumobi O, Kasasa S, Wasswa P (2016). Immunisation coverage and its determinants among children aged 12-23 months in Atakumosa-west district, Osun State Nigeria: a cross-sectional study. BMC Public Health.

[16] Oleribe O, Kumar V, Awosika-Olumo A, Taylor-Robinson SD (2017). Individual and socio-economic factors associated with childhood immunization coverage in Nigeria. Pan Afr Med J.

[17] Fatiregun AA, Okoro AO (2012). Maternal determinants of complete child immunization among children aged 12-23 months in a southern district of Nigeria. Vaccine.

[18] Sackou KJ, Oga ASS, Desquith AA, Houénou Y, Kouadio KL (2012). Couverture vaccinale complète des enfants de 12 à 59 mois et raisons de non-vaccination en milieu périurbain abidjanais en 2010. Bull Soc Pathol Exot.

[19] Ntenda PAM, Chuang KY, Tiruneh FN, Chuang YC (2017). Analysis of the effects of individual and community level factors on childhood immunization in Malawi. Vaccine.

[20] Zida-Compaore WIC, Ekouevi DK, Gbeasor-Komlanvi FA, Sewu EK, Blatome T, Gbadoe AD (2019). Immunization coverage and factors associated with incomplete vaccination in children aged 12 to 59 months in health structures in Lomé. BMC Research Notes.

[21] Landoh DE, Ouro-kavalah F, Yaya I, Kahn AL, Wasswa P, Lacle A (2016). Predictors of incomplete immunization coverage among one to five years old children in Togo. BMC Public Health.

[22] Jama AA (2020). Determinants of complete immunization coverage among children aged 11-24 months in Somalia. Int J Pediatr.

[23] Maina LC, Karanja S, Kombich J (2013). Immunization coverage and its determinants among children aged 12-23 months in a peri-urban area of Kenya. Pan Afr Med J.

